# The effect of education regarding treatment guidelines for schizophrenia and major depressive disorders on psychiatrists' hypnotic medication prescribing behavior: a multicenter study

**DOI:** 10.1186/s12888-024-05816-x

**Published:** 2024-05-29

**Authors:** Toshinori Nakamura, Ryuji Furihata, Naomi Hasegawa, Fumitoshi Kodaka, Hiroyuki Muraoka, Kayo Ichihashi, Shinichiro Ochi, Shusuke Numata, Takashi Tsuboi, Manabu Makinodan, Hitoshi Iida, Toshiaki Onitsuka, Hiroko Kashiwagi, Masahiro Takeshima, Naoki Hashimoto, Tatsuya Nagasawa, Masahide Usami, Hirotaka Yamagata, Yoshikazu Takaesu, Kenichiro Miura, Junya Matsumoto, Kazutaka Ohi, Hisashi Yamada, Hikaru Hori, Ken Inada, Koichiro Watanabe, Ryota Hashimoto, Norio Yasui-Furukori

**Affiliations:** 1 Department of Psychiatry, Shinshu University School of Medicine, 3-1-1 Asahi, Matsumoto, 390-8621 Japan; 2 Agency for Student Support and Disability Resources, Kyoto University, Yoshida-Honmachi, Kyoto, 606-8501 Japan; 3 Department of Pathology of Mental Diseases, National Institute of Mental Health, National Center of Neurology and Psychiatry, 4-1-1 Ogawahigashi, Kodaira, 187-8553 Japan; 4 Department of Psychiatry, The Jikei University School of Medicine, 3-18-13 Nishi-Shinbashi, Minato, 105-8471 Japan; 5 Department of Psychiatry, Kitasato University School of Medicine, 1-15-1 Kitazato, Sagamihara, 252-0373 Japan; 6 Department of Neuropsychiatry, University of Tokyo Hospital, 7-3-1 Hongo, Bunkyo, 113-8655 Japan; 7 Department of Neuropsychiatry, Molecules and Function, Ehime University Graduate School of Medicine, Shitsukawa, Toon, 791-0295 Japan; 8 Department of Psychiatry, Graduate School of Biomedical Science, Tokushima University, 3-8-15 Kuramoto-cho, Tokushima, 770-8503 Japan; 9 Department of Neuropsychiatry, Kyorin University School of Medicine, 6-20-2 Shinkawa, Mitaka, 181-8611 Japan; 10 Department of Psychiatry, Nara Medical University, 840 Shijo-cho, Kashihara, 634-8522 Japan; 11 Department of Psychiatry, Faculty of Medicine, Fukuoka University, 7-45-1 Nanakuma, Fukuoka, 814-0180 Japan; 12 NHO Sakakibara National Hospital, 777 Sakakibara-cho, Tsu, 514-1292 Japan; 13 Department of Forensic Psychiatry, National Center Hospital, National Center of Psychiatry and Neurology, 4-1-1 Ogawahigashi, Kodaira, 187-8551 Japan; 14 Department of Neuropsychiatry, Akita University Graduate School of Medicine, 1-1-1 Hondo, Akita, 010-8543 Japan; 15 Department of Psychiatry, Hokkaido University Graduate School of Medicine, Kita 15 Nishi 7, Sapporo, 060-8638 Japan; 16 Department of Neuropsychiatry, Kanazawa Medical University, 1-1 Daigaku, Uchinada, 920-0293 Japan; 17 Department of Child and Adolescent Psychiatry, Kohnodai Hospital, National Center for Global Health and Medicine, 1-7-1 Kohnodai, Ichikawa, 272-8516 Japan; 18 Kokoro Hospital Machida, 2140 Kamioyamadamachi, Machida, Japan; 19 Department of Neuropsychiatry, Graduate School of Medicine, University of the Ryukyus, 207 Uehara, Nishihara, 903-0215 Japan; 20 Department of Psychiatry, Gifu University Graduate School of Medicine, 1-1 Yanagido, Gifu, 501-1194 Japan; 21 Department of Neuropsychiatry, Hyogo Medical University, 1-1 Mukogawa-cho, Nishinomiya, 663-8501 Japan; 22 Department of Psychiatry, Dokkyo Medical University School of Medicine, 880 Kitakobayashi, Mibu, 321-0293 Japan

**Keywords:** Clinical practice guideline, Psychiatrist, Hypnotic medication, Schizophrenia, Major depressive disorders

## Abstract

**Background:**

To examine whether the "Effectiveness of Guideline for Dissemination and Education in psychiatric treatment (EGIUDE)" project affects the rate of prescriptions of hypnotic medication and the type of hypnotic medications prescribed among psychiatrists, for schizophrenia and major depressive disorder in Japan.

**Methods:**

The EGUIDE project is a nationwide prospective study of evidence-based clinical guidelines for schizophrenia and major depressive disorder in Japan. From 2016 to 2021, clinical and prescribing data from patients discharged from hospitals participating in the EGUIDE project were used to examine hypnotic medication prescriptions The prescribing rate of hypnotics and the prescribing rate of each type of hypnotic (benzodiazepine receptor agonist, nonbenzodiazepine receptor agonist, melatonin receptor agonist, and orexin receptor antagonist) were compared among patients who had been prescribed medication by psychiatrists participating in the EGUIDE project and patients who had been prescribed medication by nonparticipating psychiatrists. Multivariate logistic regression analysis was performed to examine the effect of the EGUIDE project on the prescription of hypnotic medications.

**Results:**

A total of 12,161 patients with schizophrenia and 6,167 patients with major depressive disorder were included. Psychiatrists participating in the EGUIDE project significantly reduced the rate of prescribing hypnotic medication and benzodiazepine receptor agonists for both schizophrenia (*P* < 0.001) and major depressive disorder (*P* < 0.001) patients.

**Conclusion:**

This is the first study to investigate the educational effects of guidelines for the treatment of psychiatric disorders on psychiatrists in terms of prescribing hypnotic medications to patients. The EGUIDE project may play an important role in reducing hypnotic medication prescription rates, particularly with respect to benzodiazepine receptor agonists. The results suggest that the EGUIDE project may result in improved therapeutic behavior.

## Background

Evidence-based medicine (EBM) involves the integration of research evidence and clinical expertise with patient intentions, behaviors, clinical situations, and environments to facilitate better decisions for patient care [1]. In clinical practice, clinical guidelines based on EBM have been developed and used to support professional-patient decision-making [2]. Meta-analyses have shown that patients with psychiatric disorders who are treated in accordance with guidelines show greater and faster recovery than patients treated with standard therapies [3]. On the other hand, it has been noted that in clinical practice, it is not uncommon for medical practices to fall outside the guidelines, and numerous medical practices diverge from established guidelines due to insufficient awareness, unfamiliarity, and disagreement with these guidelines [4].

In Japan, to implement disease treatment guidelines in clinical psychiatric practice, the Effectiveness of Guideline for Dissemination and Education in Psychiatric Treatment (EGIUDE) project was designed to educate psychiatrists about the pharmacological therapy of schizophrenia [5] and the treatment guidelines for major depressive disorder [6]. Furthermore, the EGUIDE project aimed to examine the effectiveness of guideline education by conducting follow-up studies of changes in knowledge and prescribing behavior [7]. Previous studies have shown that participation in the EGUIDE project improves participants' knowledge of and attitudes toward adhering to guidelines [7, 8] and that these effects are maintained for at least two years [9]. Moreover, participation in the EGUIDE project has been shown to be effective in improving psychiatrists' treatment-related behaviors, including antipsychotic monotherapy for schizophrenia patients and antidepressant monotherapy for major depressive disorder patients [10].

The evidence in the clinical setting regarding the use of hypnotic medication in the treatment of schizophrenia and major depressive disorder is still not clear. Although insomnia has been reported in up to 80% of patients with schizophrenia [11] and more than 90% of patients with major depressive disorders [12], in some cases as a cause and in others as a consequence [13], clinical evidence for the efficacy of hypnotic medication treatment for insomnia in patients with psychiatric disorders is limited. In this context, clinical guidelines that recommend against long-term use of prescription hypnotic medication for insomnia among patients with schizophrenia or major depressive disorder have been established [14, 15] including in Japan [5, 6]. Therefore, the EGUIDE project analyzed a large dataset of prescriptions for schizophrenia and major depressive disorder inpatients treated by physicians who had not attended a guideline course and found a high rate of prescription of hypnotic medication (55.7% of schizophrenia inpatients and 63.6% of major depressive disorder inpatients), thus revealing a potential evidence-practice gap [16].

In examining the effectiveness of EGUIDE training for prescribing hypnotic medications, it was necessary to consider the following two points in light of the current state of medical care in Japan. First, the types of hypnotic medications used in clinical practice have changed over time. Recently, orexin receptor antagonists (ORA), which are novel hypnotic medications with novel mechanisms of action, have been developed, and studies using large Japanese databases have shown that the types of hypnotic medications used have changed significantly [17, 18]. The second is the recent reform of the reimbursement system for prescribing hypnotic medications. In Japan, medical reimbursement was revised from 2012 to 2018 to optimize the prescription of hypnotic medications, and reimbursement was reduced for prescribing 3 or more sleeping pills per patient simultaneously [19].

In the present study, we used a cross-sectional survey to analyze prescription rates obtained from a database of inpatients with schizophrenia and major depressive disorder. We aimed to determine whether there is a difference in the prescribing rates of hypnotic medications between patients treated by psychiatrists who participated in the EGUIDE project and patients treated by nonparticipating psychiatrists. The objectives of this study were to determine 1) whether there is a difference in the rate of prescribing hypnotic medications between patients treated by psychiatrists who participated in the EGUIDE project and patients treated by nonparticipating psychiatrists and 2) whether there is a difference in the types of hypnotic medications prescribed for patients treated by psychiatrists who participated in the EGUIDE project and patients treated by nonparticipating psychiatrists.

## Methods

### Study design

The study is a multicenter, historical starting point cohort study; 267 medical facilities in Japan participated in the EGUIDE project between 2016 and 2021. Psychiatrists affiliated with participating facilities could self-select to participate in the EGUIDE project intervention. When psychiatrists participated in the EGUIDE project, they attended one lecture on each of the guidelines for schizophrenia and major depressive disorder. Patient data were collected from patients with schizophrenia and major depressive disorder at participating hospitals. These patient data were divided into two groups: patients treated by psychiatrists participating in the EGUIDE project (EGUIDE ( +)) and patients treated by psychiatrists not participating in the EGUIDE project (EGUIDE (-)), as described in a previous study. If both EGUIDE ( +) and EGUIDE (-) patients were assigned to the same patient, they were assigned to the EGUIDE ( +) group [10].

The EGUIDE project consisted of two days of training: one day of training on drug treatment for schizophrenia and one day of training on treatment for depression. The lecture on sleep lasted 15 to 20 min and included the identification of sleep disorders, guidance on sleep hygiene, cognitive‒behavioral therapy approach to insomnia, and points to keep in mind during drug therapy. Both disease courses lack of evidence for long-term prescribing of benzodiazepines and the risk of side effects with long-term use. The diagnosis and treatment of sleep disorders were also discussed in small-group case studies.

### Objectives

The primary outcome of the study were the rate of prescriptions of hypnotic medications among inpatients with schizophrenia and major depressive disorder and the rate of prescriptions of hypnotic medications by type of hypnotic medication.

### Participants

#### Psychiatrists

A total of 1021 psychiatrists from 267 medical facilities (48 university hospitals, 79 public hospitals, and 140 private hospitals) participating in the EGUIDE project between 2016 and 2021 attended one guideline course each on schizophrenia and major depressive disorder. Participation in the EGUIDE project was voluntary for psychiatrists from EGUIDE-participating institutions.

#### Patients

Treatment data were collected from patients with schizophrenia and patients with major depressive disorder who were discharged from the participating facilities [7, 10, 16]. Patients who were hospitalized for less than 3 days or hospitalized for laboratory purposes only were excluded. Patients were diagnosed according to DSM-5 diagnostic criteria [20], with no overlap between schizophrenia and major depressive disorder. However, the comorbidity of other diagnoses, such as substance use disorders, was not assessed.

#### Data collected

The following patient information was collected: age, gender, type of facility, whether modified electroconvulsive therapy (mECT) was administered, and whether the attending physician participated in the EGUIDE project. Data regarding the prescription of hypnotic medications, antipsychotics, anticholinergics, antidepressants, anxiolytics, and mood stabilizers/antiepileptics were collected at discharge. In addition to whether hypnotic medication was prescribed, we also identified whether benzodiazepine receptor agonists (BZA), which are classified as hypnotic medications, nonbenzodiazepine receptor agonists (nBZA), melatonin receptor agonists (MRA), or ORA, were prescribed. The classification of hypnotic medication type was determined based on previous studies [16, 21]. We investigated whether schizophrenia patients were prescribed no antipsychotics, one antipsychotic or two or more antipsychotics and whether major depressive disorder patients were prescribed no antidepressants, one antidepressant or two or more antidepressants. We assessed whether electroconvulsive therapy was administered during hospitalization.

#### Statistical analysis

Chi-square tests were used to compare categorical variables, and independent samples t tests were used to compare continuous variables between the EGUIDE ( +) and EGUIDE (-) groups. Multivariate logistic regression analysis was performed to examine the effect of the EGUIDE project on the use of hypnotic medications. The objective variables were the presence or absence of prescriptions for hypnotic medication, BZA, nBZA, MRA, and ORA. First, univariate analysis was conducted to examine factors associated with whether the patient was treated by a psychiatrist who participated in the EGUIDE project. Next, we adjusted for patient age, gender, and treatment facility (adjusted ^a^). Then, we adjusted for electroconvulsive therapy, antipsychotics, anticholinergics, antidepressants, anxiolytics, and mood stabilizers/antiepileptics (adjusted ^b^). Finally, we adjusted for the year of discharge (adjusted ^c^). Ten tests (schizophrenia, major depressive disorder, sleeping pills, BZA, nBZA, MRA, and ORA) were performed to examine the effect of participation in the EGUIDE project, so Bonferroni correction was used, and the statistical significance level was set at *P* < 0.005 (0.05/10). Statistical analyses were performed using SPSS version 29 (IBM Corp., Armonk, NY).

## Results

**Fig. 1:**
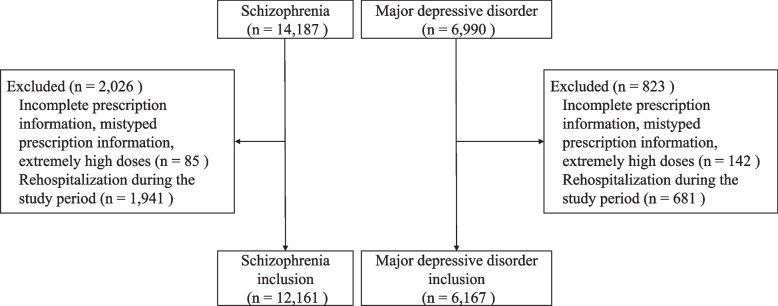
Flowchart of patient inclusion

The demographic characteristics of the EGUIDE + psychiatrists were as follows: percentage of men, 71.5%; mean age (standard deviation), 32.8 (7.0) years; and mean years of psychiatric experience at the beginning of the course, 3.9 (5.8) years. Between 2016 and 2021, a total of 14,187 patients with schizophrenia and 6,990 patients with major depressive disorder were discharged. We excluded patients with incomplete prescription information, mistyped prescription information, extremely high doses (chlorpromazine equivalent ≥ 2,000 mg/day, imipramine equivalent ≥ 350 mg/day, biperiden equivalent ≥ 7 mg/day, or diazepam equivalent ≥ 25 mg/day) (*n* = 85 for schizophrenia, *n* = 142 for major depressive disorder) or rehospitalization during the study period (*n* = 1,941 for schizophrenia and *n* = 681 for major depressive disorder). Ultimately, 12,161 patients with schizophrenia and 6,167 patients with major depressive disorder were included (Fig. 1).

Among patients with schizophrenia, in the EGUIDE ( +) and EGUIDE (-) groups, respectively, the percentage of males was 43.0% and 45.6%, and the mean age (standard deviation) was 46.08 (15.20) and 46.53 (15.83) (Table 1), and among patients with major depressive disorder, the percentage of males was 34.6% and 34.1%, and the mean age (standard deviation) was 58.14 (18.44) and 57.70 (18.38) (Table 2). The prescription rates of hypnotic medication in the EGUIDE ( +) and EGUIDE (-) groups were 50.9% and 57.5% (*P* < 0.001), respectively, for schizophrenia patients and 60.3% and 66.3% (*P* < 0.001), respectively, for major depressive disorder patients. Among patients with schizophrenia, in the EGUIDE ( +) and EGUIDE (-) groups, the respective prescription rates of BZA were 32.5% and 40.9% (*P* < 0.001), those of nBZA were 9.0% and 10.9% (*P* = 0.001), those of MRA were 4.0% and 4.1% (*P* = 0.827), those of ORA were 18.2% and 15.5% (*P* < 0.001). Among patients with major depressive disorder, the respective prescription rates of BZA were 26.1% and 35.6% (*P* < 0.001), those of nBZA were 16.4% and 18.5% (*P* = 0.034), those of MRA were 7.6% and 6.9% (*P* = 0.225), and those of ORA were 28.3% and 25.4% (*P* = 0.012). To determine whether participation in the EGUIDE project had any effect on the choice of hypnotic medication, multivariable logistic regression analysis was used to examine the effect of participation in the EGUIDE project. Multivariate logistic regression analysis to determine whether participation in the EGUIDE project had any effect on the choice of hypnotic medication revealed that EGUIDE ( +) significantly reduced the rate of prescribing hypnotic medication in both patients with schizophrenia (OR, 0.799; 95% CI, 0.736–0.866; *P* < 0.001) (Table 3) and patients with major depressive disorder (OR, 0.816; 95% CI, 0.728–0.915; *P* < 0.001) (Table 4) in all the models, adjusted or not. Next, when examined by type of hypnotic medication, the proportion of BZA prescriptions was significantly lower in schizophrenia patients (OR, 0.793; 95% CI, 0.729–0.863; *P* < 0.001) (Table 3) and those with major depressive disorder (OR, 0.771; 95% CI, 0.682–0.871; *P* < 0.001) (Table 4) in all models, adjusted or not. The proportion of nBZA prescriptions was significantly lower in schizophrenia patients in unadjusted, adjusted ^a^, and adjusted ^b^, and the proportion of ORA prescriptions was significantly higher in schizophrenia in patients unadjusted, adjusted ^a^, and adjusted ^b^ (Table 3).

**Table 1:** Characteristics of the patients with schizophrenia

	EGUIDE ( +)		EGUIDE (-)		*P*-value
	n	%	n	%	
Number of patients	4481		7680		
Age, mean (SD) years	46.08	15.20	46.53	15.83	0.125
Sex					0.005
Men	1927	43.0%	3503	45.6%	
Women	2554	57.0%	4177	54.4%	
Type of facilities					< 0.001
University hospitals	1598	35.7%	2306	30.0%	
Public hospitals	1528	34.1%	3031	39.5%	
Private hospitals	1355	30.2%	2343	30.5%	
mECT treatment					< 0.001
Yes	333	7.4%	443	5.8%	
No	4148	92.6%	7237	94.2%	
Antipsychotics					< 0.001
2 or more	1815	40.5%	3432	44.7%	
1	2618	58.4%	4158	54.1%	
0	48	1.1%	90	1.2%	
Anticholinergic drugs					< 0.001
Yes	1037	23.1%	2208	28.8%	
No	3444	76.9%	5472	71.3%	
Antidepressants					0.013
Yes	327	7.3%	658	8.6%	
No	4154	92.7%	7022	91.4%	
Anxiolytics					< 0.001
Yes	1086	24.2%	2175	28.3%	
No	3395	75.8%	5505	71.7%	
Mood stabilizers/Antiepileptic drugs					< 0.001
Yes	1027	22.9%	1995	26.0%	
No	3454	77.1%	5685	74.0%	
Year the data was obtained					< 0.001
2016	0	0.0%	1126	14.7%	
2017	488	10.9%	1149	15.0%	
2018	687	15.3%	1483	19.3%	
2019	944	21.1%	1643	21.4%	
2020	1064	23.7%	1195	15.6%	
2021	1298	29.0%	1084	14.1%	
Hypnotic medication use					< 0.001
No	2199	49.1%	3266	42.5%	
Any hypnotic medication	2282	50.9%	4414	57.5%	
Types of hypnotic medication					
BZA	1456	32.5%	3139	40.9%	< 0.001
nBZA	405	9.0%	834	10.9%	0.001
MRA	179	4.0%	313	4.1%	0.827
ORA	816	18.2%	1192	15.5%	< 0.001

EGUIDE ( +), patients treated by psychiatrists who participated in the EGUIDE project; EGUIDE (-), patients treated by psychiatrists who did not participate in the EGUIDE project; SD, standard deviation

Abbrevations: *mECT* modified electroconvulsive therapy, *BZA* benzodiazepine receptor agonists, *nBZA* nonbenzodiazepine receptor agonists, *MRA* melatonin receptor agonists, *ORA* orexin receptor antagonists

**Table 2:** Characteristics of the patients with major depressive disorder

	EGUIDE ( +)		EGUIDE (-)		*P*-value
	n	%	n	%	
Number of patients	2452		3715		
Age, mean (SD) years	58.14	18.44	57.70	18.38	0.360
Sex					0.714
Men	848	34.6%	1268	34.1%	
Women	1604	65.4%	2447	65.9%	
Type of facilities					< 0.001
University hospitals	1299	53.0%	1911	51.4%	
Public hospitals	546	22.3%	988	26.6%	
Private hospitals	607	24.8%	816	22.0%	
mECT treatment					0.454
Yes	368	15.0%	532	14.3%	
No	2084	85.0%	3183	85.7%	
Antipsychotics					0.758
Yes	1252	51.1%	1882	50.7%	
No	1200	48.9%	1833	49.3%	
Anticholinergic drugs					0.067
Yes	47	1.9%	98	2.6%	
No	2405	98.1%	3617	97.4%	
Antidepressants					< 0.001
2 or more	514	21.0%	971	26.1%	
1	1688	68.8%	2318	62.4%	
0	250	10.2%	426	11.5%	
Anxiolytics					< 0.001
Yes	706	28.8%	1298	34.9%	
No	1746	71.2%	2417	65.1%	
Mood stabilizers/Antiepileptic drugs					< 0.001
Yes	257	10.5%	531	14.3%	
No	2195	89.5%	3184	85.7%	
Year the data was obtained					< 0.001
2016	0	0.0%	552	14.9%	
2017	232	9.5%	603	16.2%	
2018	406	16.6%	641	17.3%	
2019	509	20.8%	732	19.7%	
2020	539	22.0%	570	15.3%	
2021	766	31.2%	617	16.6%	
Hypnotic medication use					< 0.001
No	959	39.1%	1253	33.7%	
Any hypnotic medication	1493	60.9%	2462	66.3%	
Types of hypnotic medication					
BZA	641	26.1%	1322	35.6%	< 0.001
nBZA	403	16.4%	689	18.5%	0.034
MRA	189	7.7%	256	6.9%	0.225
ORA	694	28.3%	944	25.4%	0.012

EGUIDE ( +), patients treated by psychiatrists who participated in the EGUIDE project; EGUIDE (-), patients treated by psychiatrists who did not participate in the EGUIDE project; SD, standard deviation

Abbrevations: *mECT* modified electroconvulsive therapy, *BZA* benzodiazepine receptor agonists, *nBZA* nonbenzodiazepine receptor agonists, *MRA* melatonin receptor agonists, *ORA* orexin receptor antagonists

**Table 3:** Logistic regression analysis showing the adjusted effects (odds ratios with 95% CIs) between the EGUIDE project and hypnotic medication use in patients with schizophrenia (*n* = 12,161)

	Unadjusted				Adjusted^a^				Adjusted^b^				Adjusted^c^			
	OR	95%CI		*P*-value	OR	95%CI		*P*-value	OR	95%CI		*P*-value	OR	95%CI		*P*-value
Hypnotic medication use																
Any hypnotic medication	0.768	0.713	0.827	< 0.001*	0.767	0.712	0.827	< 0.001*	0.819	0.758	0.885	< 0.001*	0.799	0.736	0.866	< 0.001*
Types of hypnotic medication																
BZA	0.696	0.644	0.752	< 0.001*	0.698	0.646	0.754	< 0.001*	0.748	0.690	0.811	< 0.001*	0.793	0.729	0.863	< 0.001*
nBZA	0.816	0.720	0.924	0.001*	0.807	0.712	0.915	< 0.001*	0.834	0.735	0.946	0.005*	0.859	0.752	0.981	0.025
MRA	0.979	0.812	1.181	0.827	1.002	0.830	1.209	0.986	0.996	0.824	1.204	0.967	1.049	0.858	1.282	0.644
ORA	1.212	1.099	1.336	< 0.001*	1.217	1.104	1.343	< 0.001*	1.231	1.114	1.359	< 0.001*	0.977	0.880	1.084	0.658

Abbrevations: *BZA* benzodiazepine receptor agonists, *nBZA* nonbenzodiazepine receptor agonists, *MRA* melatonin receptor agonists, *ORA* orexin receptor antagonists

^a^Adjusted for patients age, sex, and type of facilities

^b^Adjusted for patients age, sex, type of facilities, mECT, antipsychotics (2 or more, one, no), anticholinergics, antidepressants, anxiolytic, and mood stabilizers/antiepileptic drugs

^c^Adjusted for patients age, sex, type of facilities, mECT, antipsychotics (2 or more, one, no), anticholinergics, antidepressants, anxiolytic, mood stabilizers/antiepileptic drugs, and years

^*^Significant differences (*P* < 0.005)

**Table 4:** Logistic regression analysis showing the adjusted effects (odds ratios with 95% CIs) between the EGUIDE project and hypnotic medication use in patients with major depressive disorder (*n* = 6,167)

	Unadjusted				Adjusted ^a^				Adjusted ^b^				Adjusted ^c^			
	OR	95%CI		*P*-value	OR	95%CI		*P*-value	OR	95%CI		*P*-value	OR	95%CI		*P*-value
Hypnotic medication use																
Any hypnotic medication	0.792	0.713	0.881	< 0.001*	0.791	0.712	0.880	< 0.001*	0.832	0.747	0.927	< 0.001*	0.816	0.728	0.915	< 0.001*
Types of hypnotic medication																
BZA	0.641	0.573	0.717	< 0.001*	0.640	0.571	0.716	< 0.001*	0.676	0.602	0.760	< 0.001*	0.771	0.682	0.871	< 0.001*
nBZA	0.864	0.755	0.989	0.034	0.861	0.752	0.986	0.030	0.883	0.770	1.012	0.074	0.905	0.784	1.046	0.176
MRA	1.128	0.928	1.372	0.225	1.136	0.934	1.383	0.202	1.144	0.939	1.395	0.182	1.120	0.909	1.380	0.288
ORA	1.159	1.033	1.301	0.012	1.159	1.033	1.301	0.012	1.172	1.044	1.317	0.007	0.976	0.863	1.104	0.696

Abbrevations: *BZA* benzodiazepine receptor agonists, *nBZA* nonbenzodiazepine receptor agonists, *MRA* melatonin receptor agonists, *ORA* orexin receptor antagonists

^a^Adjusted for patients age, sex, and type of facilities

^b^Adjusted for patients age, sex, type of facilities, mECT, antipsychotics, anticholinergics, antidepressants (2 or more, one, no), anxiolytic, and mood stabilizers/antiepileptic drugs

^c^Adjusted for patients age, sex, type of facilities, mECT, antipsychotics, anticholinergics, antidepressants (2 or more, one, no), anxiolytic, mood stabilizers/antiepileptic drugs, and years

^*^Significant differences (*P* < 0.005)

## Discussion

This is the first study to investigate the educational effects of guidelines for the treatment of psychiatric disorders in terms of prescribing hypnotic medications for patients with schizophrenia and major depressive disorder on psychiatrists in Japan.

The results of this study revealed that psychiatrists who attended the EGUIDE project prescribed hypnotic medication at significantly lower rates, including significantly lower rates of prescribing BZA. Few studies have investigated the actual prescription rate of hypnotic medications in the treatment of psychiatric disorders. In clinical studies examining patients with schizophrenia, hypnotic medications were prescribed in 11.2% to 55.7% of patients [16, 22], and in studies of major depressive disorder patients, they were prescribed in 29.6% to 63.6% of patients [16, 23]. Although insomnia is often a persistent condition [24] and several novel mechanisms of action of hypnotic medications have recently been shown to be effective and safe for long-term use in appropriate clinical settings [25], long-term pharmacotherapy with BZA, which is often used in real-world clinical practice, is associated with falls, delirium, cognitive decline, respiratory depression, dependence and withdrawal [26], as well as the risk of being used in suicide attempts [27].

There are several possible methods to optimize the prescription of hypnotic medications. First, postgraduate education for psychiatrists working at the front line of treatment on basic medical treatment methods and treatment techniques for psychiatric patients with sleep problems is considered an important option to promote behavioral change among psychiatrists. It has been previously reported that participation in the EGUIDE project increased psychiatrists' expertise and changed their practice behavior [7, 9, 10]. The second method is to provide more specialized sleep treatment. Cognitive behavioral therapy for insomnia (CBT-I) is considered an important therapeutic option for chronic insomnia, and there is some evidence that it leads to a reduction in the use of hypnotic medications [28]. However, in Japan, as in other Western countries, access to CBT-I is limited, and it is not commonly provided in primary care [29]. The third method is to revise medical fees. In Japan, reimbursement was revised three times between 2012 and 2018 to promote the appropriate use of hypnotic medications, reducing reimbursement for prescribing three or more hypnotic medications in a single prescription [19]. This revision was shown to reduce the rate of polypharmacy, but the effect on long-term prescriptions is not clear [30].The introduction of secure prescription pads in France serves as another example of regulatory intervention. This initiative notably reduced zolpidem exposure among chronic users, extensively altered prescribing behaviors for other sedatives, and increased general practitioners’ understanding of hypnotic medication risks [31, 32]. Since the present study lacked clinical information on the importance of patients' psychiatric symptoms and insomnia symptoms, it is not possible to judge whether the treatment is appropriate or not only because of the lower rate of prescription of hypnotic medication. However, because long-term prescribing of hypnotic medication is not recommended in treatment guidelines in Japan [5, 6] or Western countries [14, 15] and because administrative-level measures against prescribing of hypnotic medication are needed in the Japanese medical field [30], the prescribing behavior of physicians who participate in education programs should be more appropriate, thereby improving the evidence-practice gap.

The current survey showed that BZA prescriptions are less common among psychiatrists who participate in the EGUIDE project. There are two possible interpretations for this. The first is a possible effect of EGUIDE training. Although the EGUIDE training does not recommend prescribing a specific type of hypnotic medication, as the treatment guidelines in Japan [5, 6] do not change the recommendation according to the type of hypnotic medication, it does alert participants to the side effects of BZA in general and to the fact that long-term, indolent treatment may not be useful in the treatment of patients. EGUIDE training also increased clinical behavior in accordance with treatment guidelines, which were maintained two years later [9]. Therefore, prescribing behavior may be changing among EGUIDE ( +) psychiatrists toward avoiding prescribing BZA more as a hypnotic medication. Second, it may reflect changes in the prescription of hypnotic medications over time. A study using the Japanese clime database reported a significant decrease in BZA and an increase in ORA from 2010 to 2019 [17]. However, this study took this into account and adjusted for the year in which the data were obtained as a covariate. While ORA lost significance at adjusted ^c^ and was affected by the year, BZA had significant differences at adjusted ^c^ as well. Therefore, it is unlikely that group differences were observed solely due to changes over time. Therefore, it is difficult to assume that differences between groups were found only by changes over time.

The current study has several limitations. First, this study did not use random assignment regarding participation in the EGUIDE project. Therefore, participants in the EGUIDE project included many who were highly motivated to practice EBM and EGUIDE ( +) psychiatrists and EGUIDE (-) psychiatrists may not treat the same type of patient. These factors may have led to selection bias. It is known that EGUIDE (-) practitioners are investigated for prescribing patterns, as EGUIDE facilities investigate prescribing patterns. However, EGUIDE ( +) may have led to a reduction in the prescription rate of hypnotic medications, as it is clearly explained at the time of attending EGUIDE that prescribing patterns would be investigated. Second, the study was cross-sectional with prescribing patterns assessed at one point in time. It was impossible to assess whether guideline education affects the long-term prescription of hypnotic medications. Third, there was no information on gender, age, or years of psychiatric experience in the EGUIDE(-) group, and information was shared between those who took the course and those who did not take the course within the same facility, which may have contaminated the effectiveness of the intervention. In the future, it may be necessary to collect data from facilities that do not participate in the EGUIDE project to make comparisons between facilities. Fourth, clinical data on symptoms and disease severity of treated patients were not assessed using rating scales. Future studies should use clinical symptom measurement scales, including observer-rated or self-rated scales, such as the Positive and Negative Syndrome Scale, the Beck Depression Inventory, or the RU-SATED, a self- rated sleep scale for the measurement of multidimensional sleep health　[33, 34]. Fifth, other confounding factors, such as length of hospitalization and number of patients with substance use disorders, that were not adjusted for in the present study may have influenced the results.

## Conclusions

This study investigated the effects of educating psychiatrists on disease treatment guidelines based on the actual prescribing of hypnotic medication for the patients they treated and found that psychiatrists who participated in the EGUIDE project had a significantly lower rate of prescribing hypnotic medication and a significantly lower rate of BZA prescriptions according to the type of hypnotic medication. The results suggest that educational activities involving evidence-based treatment guidelines may play an important role in the postgraduate education of psychiatrists to optimize therapeutic behavior.

## Data Availability

The datasets analyzed during the current study are not publicly available due to informed consent, which has not been obtained for the release of raw data but is available from the corresponding author upon reasonable request.

## Abbreviations

BZA: Benzodiazepine receptor agonist
CBT-I: Cognitive behavioral therapy for insomnia
EBM: Evidence-based medicine
EGUIDE: Effectiveness of Guideline for Dissemination and Education in psychiatric treatment
mECT: Modified electroconvulsive therapy
MRA: Melatonin receptor agonists
nBZA: Nonbenzodiazepine receptor agonists
ORA: Orexin receptor antagonists
